# Permissive underfeeding versus target enteral feeding in adult critically ill patients (PermiT Trial): a study protocol of a multicenter randomized controlled trial

**DOI:** 10.1186/1745-6215-13-191

**Published:** 2012-10-12

**Authors:** Yaseen M Arabi, Samir H Haddad, Abdulaziz S Aldawood, Hasan M Al-Dorzi, Hani M Tamim, Maram Sakkijha, Gwynne Jones, Lauralyn McIntyre, Sangeeta Mehta, Othman Solaiman, Musharaf Sadat, Lara Afesh, Bushra Sami

**Affiliations:** 1 King Saud bin Abdulaziz University for Health Sciences, Riyadh, Saudi Arabia; 2 King Abdulaziz Medical City, Riyadh, Saudi Arabia; 3 King Abdullah International Medical Research Center, Riyadh, Saudi Arabia; 4 Ottawa Hospital Research Institute, Ottawa, Canada; 5 Mount Sinai Hospital, Toronto, Canada; 6 King Faisal Specialist Hospital and Research Center, Riyadh, Saudi Arabia; 7 Chairman Intensive Care Department, Medical Director Respiratory Services, Associate Professor, College of Medicine, King Abdul Aziz Medical City, King Saud bin Abdulaziz University for Health Sciences, 1425, PO Box 22490, Riyadh, 11426, Kingdom of Saudi Arabia

**Keywords:** Enteral nutrition, Intensive Care Units, Caloric restriction, Infections, Insulin, Mortality

## Abstract

**Background:**

Nutritional support is an essential part of the management of critically ill patients. However, optimal caloric intake has not been systematically evaluated. We aim to compare two strategies of enteral feeding: permissive underfeeding versus target feeding.

**Method/Design:**

This is an international multi-center randomized controlled trial in critically ill medical- surgical adult patients. Using a centralized allocation, 862 patients will be randomized to permissive underfeeding or target feeding. Patients in the permissive group receive 50% (acceptable range is 40% to 60%) of the calculated caloric requirement, while those in the targeted group receive 100% (acceptable range 70% to 100%) of the calculated caloric requirement. The primary outcome is 90-day all-cause mortality. Secondary outcomes include ICU and hospital mortality, 28-day, and 180-day mortality as well as health care-associated infections, organ failure, and length of stay in the ICU and hospital. The trial has 80% power to detect an 8% absolute reduction in 90-day mortality assuming a baseline risk of death of 25% at an alpha level of 0.05.

**Discussion:**

Patient recruitment started in November 2009 and is currently active in five centers. The Data Monitoring Committee advised continuation of the trial after the first interim analysis. The study is expected to finish by November 2013.

**Trial registration:**

Current Controlled Trials ISRCTN68144998

## Background

Nutritional support is an essential part of the management of critically ill patients [1]. Although clinical trials have resolved the ‘when to start feeding’ question [1], which is within 24 to 48 hours after admission to the ICU, there is little evidence to answer the ‘how much’ question. During critical illness, the metabolic rate is increased and complex fuel alterations occur. Fuel choice is altered from predominantly fat to glucose oxidation, thus glycolysis increases enormously. Proteolysis and lipolysis are also augmented [2]. In a healthy person, exogenously supplied calories from fat or carbohydrate will suppress lipolysis and proteolysis [2]. However, this suppression is blunted by acute inflammatory illness [2]. In fact, excess caloric provision is increasingly perceived as having detrimental effects, particularly on mitochondria [3], since it leads to increased oxygen radical production in addition to wasting fuel [3].

In 1997, a review concluded that there was insufficient data from methodologically sound studies to permit firm recommendations about the optimal amount of nutritional support for critically ill patients [4]. Likewise, the 2003 Canadian clinical practice guidelines concluded that whereas early enteral nutrition was recommended in critically ill patients, there were insufficient data to make recommendations on the dose of enteral feeding except for head-injured patients [5]. Even in head injury patients, the recommendations for optimizing delivery of nutrients were based on only one randomized controlled trial(RCT) [5].

The optimal caloric intake in the critically ill remains controversial. This controversy has been heightened with recent evidence suggesting harm and other studies suggesting benefit with the use of lower caloric intake in the critically ill. In a single-center RCT of 82 head-injured patients, the effect of ‘early-enhanced’ enteral nutrition (59.2% of caloric goal) was compared with standard nutrition (36.8% of caloric goal). At three months, the enhanced nutrition group had a trend towards better neurological outcome in comparison to the standard nutrition group and fewer overall complications, including infections [6]. However, there were no differences in neurological outcome at six months or in mortality [6]. The multicenter cluster-randomized Algorithms for Critical Care Enteral and Parenteral Therapy (ACCEPT) trial investigated the impact of implementing evidence-based feeding algorithms on nutrition practices and patient outcomes in the ICU [7]. Patients in the intervention group received more calories per day than those in the control group (1,264 kcal versus 998 kcal, *P* = 0.31) and had a significantly shorter hospital length of stay (LOS) with a trend towards reduced mortality. Rubinson *et al.* showed that very low caloric intake (<25% of the average daily recommended calories) was associated with an increased risk of health care-associated bloodstream infections [8]. A single-center RCT of 28 patients showed that lower energy deficits were associated with a decreased need for renal replacement therapy [9].

There is also evidence to support outcome benefit with lower than standard targeted feeding goals. In a cohort study, Krishnan *et al.* found that moderate caloric intake (33% to 65% of the recommended American College of Chest Physicians (ACCP) targets) was associated with better clinical outcomes including reduced mechanical ventilation duration, ICU LOS, as well as hospital mortality, when compared with higher caloric intake [10]. However, this study did not adjust for glucose control that has been found to influence outcomes in ICU patients. Dickerson *et al.* studied 40 critically ill, obese patients and found that patients who received fewer calories (<20 kcal/kg versus ≥20 kcal/kg) had decreased ICU LOS (*P* <0.03), reduced duration of antibiotic therapy (*P* <0.03), and a trend towards decreased mechanical ventilation duration (*P* = 0.09) [11]. In a single-center quasi-RCT, 150 mechanically ventilated medical patients were administered ‘early aggressive’ feeding or ‘late’ feeding [12]. The late group received fewer calories (629 ± 575 kcal versus 2,370 ± 2,000 kcal, *P* <0.001) and had a lower incidence of ventilator-associated pneumonia (30.7% versus 49.3%, *P* = 0.02), less *Clostridium difficile* induced diarrhea (4.0% versus 13.3%, *P* = 0.04), as well as shorter ICU LOS (9.8 ± 7.4 versus13.6 ± 14.2 days, *P* = 0.04) and hospital LOS (16.7 ±12.5 days versus 22.9 ± 19.7 days, *P* = 0.02) compared with the early feeding group [12]. An RCT with 200 mechanically ventilated patients and a second RCT with 1,000 patients with acute lung injury showed that a strategy of initial trophic enteral feeding (10 mL/hour) compared with standard targeted enteral feeding goals for up to six days reduced gastrointestinal tolerance, but did not improve ventilator-free days or 60-day mortality [13,14]. The intervention arms of these two RCTs instituted very low trophic feeding in critically ill patients with acute lung injury. In contrast, an RCT of 240 general medical-surgical mechanically ventilated patients conducted by our investigative team compared the efficacy of a moderate hypo caloric (60% to 70% of calculated caloric requirement) versus a eucaloric (90% to 100%) diet [15]. The endpoints of ICU, 28-day and 90-day mortality were not statistically different (17.5%, versus 21.7%, *P* = 0.42; 18.3% versus 23.3%, *P* = 0.34 and 31.0% versus 39.3%, *P* = 0.19, respectively) [15]. However, hospital mortality was reduced in the hypo caloric diet group (30% versus 42.5%, *P* = 0.04), and there was a trend towards shorter ICU LOS [15]. In contrast to very low caloric or trophic enteral feeding, it remains unclear whether a moderate caloric intake may improve survival and reduce morbidity.

### Rationale for the study

There is a lack of conclusive evidence regarding the caloric intake dose required for enteral feeding in critically ill patients. The available literature reports mainly on observational studies that do not provide the best evidence and existing RCTs are either conducted on specific patient populations or are not conclusive. The objective of our Permissive Underfeeding versus Target Enteral Feeding in Adult Critically Ill Patients (PermiT) trial is to assess the effect of permissive underfeeding versus target feeding on 90-day mortality in adult, medical-surgical critically ill patients.

## Methods/design

### Study population, inclusion and exclusion criteria

All patients admitted to the participating ICUs are screened daily for study eligibility. Patients are deemed eligible for this study if they are ≥18-year old, receiving enteral feeding, and expected to remain in ICU ≥72 hours (Time Zero would be the time of ICU admission). Exclusion criteria are: lack of commitment to ongoing life support, brain death, pre-existing condition with expected six month mortality >50%, enteral feeding not started within 48 hours of ICU admission, parenteral nutrition (PN), oral feeding, previous enrollment in this study within the same hospital admission, pregnancy, post liver transplantation, post cardiac arrest, burns, prisoners, and receipt of vasopressors (except dobutamine) at the following doses: norepinephrine >0.4 μg/kg/min, epinephrine >0.4 μg/kg/min, dopamine >20 μg/kg/min, phenylephrine >300 μg/min, vasopressin >0.04 unit/min or half of these doses for patients receiving two or more vasopressors. Patients who are initially ineligible are reassessed multiple times for eligibility within the randomization window of 48 hours.

Eligible patients are not randomized (eligible non-randomized) if their substitute decision makers decline consent, the research team is unable to get consent within 48 hours of eligibility, the treating ICU physician does not agree with the patient being enrolled in the study or if the patient is already enrolled in a trial of a similar nature or outcome.

### Randomization

PermiT is a multicenter open-label international RCT. The patients who meet all inclusion with no exclusion criteria are randomized into one of the two intervention arms: the permissive underfeeding group or target feeding group. Randomization is based on computer-generated random permuted blocks of four and is stratified by center and follows a concealed process using sealed and numbered envelopes that allocate the patient to either of the two arms of the study.

Patients may be randomized into PermiT only once unless they were discharged from the hospital and were re-admitted beyond 180 days of the first enrollment. The study does not allow cross-overs and if any occur, they will be reported as protocol violations.

### Informed consent

The research coordinator and/or physician investigator explains the objectives of this study and its potential risks and benefits to the patient when possible or more commonly to his/her surrogate decision maker. Once the patient or his/her surrogate decision maker agrees, a witnessed written consent is obtained. The patient or surrogate can withdraw from the study at any time without impact on treatment. A screening log indicating why eligible patients were not randomized is recorded at each of the participating centers and reported back to the coordinating center on a three-month basis.

### Trial interventions and nutrition related co-interventions

Patients in the permissive underfeeding group receive 50% of the calculated caloric requirement (with an acceptable range of 40% to 60%) and those in the target feeding group receive100% of the calculated caloric requirement (with an acceptable range of 70% to 100%). These targets were selected to maintain moderate caloric intake in the permissive underfeeding group yet to achieve a larger separation in caloric intake between the two groups compared to our previous trial [15]. Participating centers may use their own standard feeding protocols to achieve the caloric intake for both arms of the study. The coordinating center uses a feeding protocol that has been published earlier [16]. Head of bed elevation, minimizing discontinuation of enteral feeding during diagnostic tests, nursing care or routine bedside procedures are followed according to the most current Society of Critical Care Medicine (SCCM) and American Society for Parenteral and Enteral Nutrition (ASPEN) clinical practice guidelines [17]. After radiographic confirmation of tube placement (gastric or post-pyloric), feeding is started at 30 ml/hour and advanced by 10 ml/hour every four hours. To achieve the prescribed enteral feeding, daily targets are used in addition to the hourly rate [18]. The protocol does not provide recommendations for the type of feeding tube (large-bore or small-bore nasogastric tube with or without guide wire). For patients fed with gastric tubes, the residuals are checked every four hours, and for post-pyloric tubes only if there are signs of feeding intolerance such as vomiting, abdominal distention or ileus. For small size tubes that do not permit aspiration, a second larger size tube may be placed into the stomach to aspirate the gastric residual. The protocol provides instructions on the management of residuals greater than 200 ml. Prokinetic agents and post-pyloric tubes for feeding intolerance are used at the discretion of the treating physicians. The decision to introduce PN is also at the discretion of the treating team. Our PermiT study recommends following the SCCM/ASPEN clinical practice guidelines, which considers PN for both the targeted and permissive underfeeding groups if caloric requirements are not met after 7 to 10 days by enteral route alone [17].

#### Enteral formulations

The type of enteral formulations used is not directed by the study protocol and is left to the combined decision of the treating physician and the clinical dietitian as long as caloric targets are met. The types of formulae are classified into non-specialized (Ensure, Ensure Plus, Jevity (1.0 to 1.2), Osmolite, Resourse and Resourse Plus (from Nestle, Novartis and Abbott, Laboratories,) or specialized (Nepro, Pulmocare, Renal Nova Source, Glucerna, Nutren Hepatic, Suplena, Vivonex Plus and Peptamen (1.0, 1.2, and 1.5), and Promote (from Nestle, Novartis and Abbott Laboratories)). Beneprotein formula (from Novartis) is used to supplement protein. The choice of using either immune-modulating or non-immune modulating formulations is left to the discretion of participating centers. Suggestions on selection of enteral formulations depending on patients’ health status are outlined in Table 1 and consistent with the SCCM/ASPEN clinical practice guidelines [17]. 

**Table 1:** Guidelines for selection of enteral feeding formulation

**Diagnostic category**	**Enteral feeding formulation**
Major elective surgery, trauma, burns, headand neck cancer, and critically ill patients on mechanical ventilation (being cautious in patients with severe sepsis)	Immune-modulating enteral formulations (arginine, glutamine, nucleic acid, omega-3 fatty acids, and antioxidants). Grade A (surgical) Grade B (medical).
Acute lung injury/acute respiratory distress syndrome	Enteral formulation characterized by an anti-inflammatory lipid profile (that is, omega-3 fish oils, borage oil) and antioxidants (grade A).
All critically ill patients receiving specialized nutrition therapy	Antioxidant vitamins (including vitamins E and ascorbic acid) and trace minerals (specifically including selenium, zinc and copper).
Burn, trauma, and mixed ICU patients	Enteral glutamine to an enteral nutrition regimen.
Pulmonary failure	High-lipid low carbohydrate formulations are not recommended. Fluid-restricted calorically dense formulations should be considered.
Renal failure	Standard enteral formulations and standard ICU recommendations for protein and calorie.

#### Calculation of caloric requirements

The caloric requirement is calculated by the clinical dietitian on study day 1 and then recalculated on study day 7 ± 1 using different predictive equations [see Additional file 1, Appendix A] depending on body mass index (BMI) and whether the patient is breathing spontaneously or is mechanically ventilated [19-22]. The Penn State Equation is used for mechanically ventilated patients with BMI <30, and the Ireton-Jones, 1992 Equation is used for mechanically ventilated patients with BMI ≥30 and for spontaneously breathing patients.

#### Calculation of protein requirement

Both study groups receive their daily protein requirements. Protein requirements are based on nutritional status, degree of stress of disease or injury and physiological capability to metabolize protein. For the metabolically stressed patient, current SCCM/ASPEN clinical practice guidelines recommend 1.2 to 1.5 g of protein/kg/day [17,23].

#### Blood glucose management

In both groups, the target blood glucose level is 4.4 to 10 mmol/L (80 to 180 mg/dL). Study centers may use their own insulin therapy protocols to achieve this blood glucose target.

#### Other co-interventions

The use of propofol for sedation, which adds to the caloric intake, is administered according to the treating team. Each 1 ml of propofol (propofol 1% or 2% Fresenius) provides 1.1 Kcal. To reduce the caloric intake from propofol, the following are recommended: 1) minimize the use of propofol; 2) use propofol for short term sedation (up to 72 hours) as per SCCM guidelines [17]; and 3) limit caloric intake from propofol to ≤500 kcal/day for patients who weigh >70 kg and ≤300 kcal/day for patients who weigh ≤70 kg. The use of dextrose-containing fluids is according to the treating team and the insulin protocol. However, the caloric contribution of dextrose and propofol is accounted for by adjusting the enteral nutrition caloric intake on a daily basis by the study team.

To account for the volume difference between the two groups, patients in the permissive underfeeding receive 2 mL/kg of normal saline or water every four hours (based on an estimated difference in the administered volume of 30 to 40 ml/hour between the two intervention groups) by the same enteral route unless otherwise specified by the physician. All receive multivitamins (including vitamin E, vitamin C) and trace elements (selenium, zinc and copper) on a daily basis. All other co-interventions are left to the discretion of the treating team.

#### Duration of the intervention

The allocated intervention is continued until any of the following criteria is met: 1) a maximum of 14 days on the study feeding protocol; 2) ICU discharge; 3) ICU mortality or decision to withhold feeds as a part of a palliative care plan; or 4) oral feeding started and tolerated for >24 hours.

### Data collection, frequency and duration of follow-up following recruitment

At baseline for all randomized patients, data obtained include demographics, presence of co-morbidities such as chronic health problems, medication use prior to admission, Acute Physiology and Chronic Health Evaluation Scores (APACHE) II [24] and III [25] and presence of sepsis upon admission. Nutritional data (total calories and total protein), laboratory data (morning blood glucose, total insulin, hypoglycemic episodes, creatinine, potassium, magnesium and phosphorous), fluid input–output and stool frequency are recorded on a daily basis until a maximum of 14 days of ICU stay, ICU discharge or ICU mortality, whichever comes first. Data about healthcare-associated infections are also collected until two days after ICU discharge.

### Outcome measures

The primary outcome is 90-day all-cause mortality. Secondary outcomes include ICU, hospital, 28-day and 180-day mortality rates and Sequential Organ Failure Assessment (SOFA) scores (on days 1, 3, 7, 14, 21 and 28). Tertiary outcomes include ICU and hospital LOS, hypoglycemia, hypokalemia, hypophosphatemia, hypomagnesemia, health care-associated infections [26], refeeding syndrome [27], and diarrhea [28]. Definitions of different outcomes are outlined in Additional file 1: Appendix B. Hospital mortality is censored at one year from the date of enrollment. Vital status at day 90 and day 180 are recorded if needed by telephone interview with the patient or his/her surrogate decision maker.

### Administrative and ethical aspects

The study sponsor is KAIMRC. The coordinating study center is the Intensive Care Department at King Saud bin Abdulaziz University for Health Sciences (KSAUHS)/King Abdullah International Medical Research Center (KAIMRC) in Riyadh, Saudi Arabia. Participating centers include the Ottawa Hospital (General and Civic campuses), Ottawa, Canada; Mount Sinai Hospital, Toronto, Canada; and King Faisal Specialist Hospital and Research Center, Riyadh, Saudi Arabia.

The study is being conducted in accordance with the International Conference on Harmonization-Good Clinical Practice (ICH-GCP) guidelines. The study protocol and the informed consent have been approved by the KSAUHS/KAIMRC Institutional Review Board (IRB) (approval number IRBC/109/10) followed by the respective IRBs of all the participating centers.

Several measures are taken to ensure optimal compliance with the study protocols. Before launching the study, ICU physicians, nurses and dietitians attended training sessions with special emphasis on adjustment of feeding to achieve the target caloric intake. The Steering Committee (YMA, SHH, ASA, HMD, and HMT) is responsible for overall management of the study including providing central guidance to support participating centers for protocol adherence, addressing challenges with protocol implementation, formulating the analysis plan, reviewing and interpreting data and preparing the manuscript. The average caloric intake is compared between the two groups on a regular basis every two months and is stratified by center. Feedback is provided to each center to further improve adherence to caloric targets, if needed. The sponsor has appointed an independent monitor who ensures that the trial is conducted in accordance with the ethical principles of the Declaration of Helsinki and the ICH-GCP guidelines. The monitor has direct access to case report forms, the source document and the trial master file at the coordinating study center. The study also has an independent Data Monitoring Committee (DMC) which is responsible for overseeing the safety of study patients, monitoring efficacy and protocol adherence and making recommendations to continue or terminate the study based on safety analysis results. The DMC is composed of three members who are not involved in the planning or execution of the study.

### Analytical plan

#### Sample size

**Figure 1:**
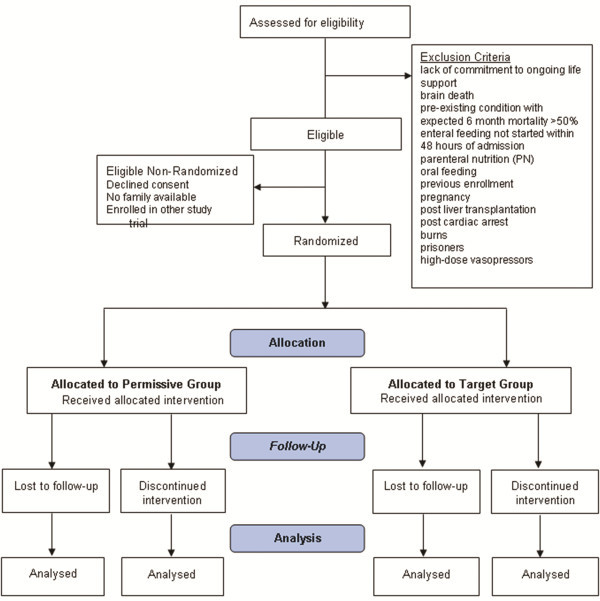
Flow diagram.

In our recent RCT [15], we observed an absolute risk reduction in hospital mortality of 8% (95% CI 0.4% to 25%). Based on an estimated 90-day mortality of 25% and using a power of 80% and an alpha of 0.05, the sample required to demonstrate a decrease in mortality of 8% was 431 in each group (a total of 862 patients). The flow of patients through the study will be displayed in a ‘CONSORT’ diagram (Figure 1). 

#### Statistical and analytical plan

Patients will be analyzed according to the intention-to-treat principle, that is, all randomized patients will be analyzed in the groups to which they were originally allocated. Baseline characteristics will be summarized by carrying out univariate analyses. Categorical variables will be presented as numbers and percents, whereas the continuous variables will be presented as mean and standard deviations. The primary and secondary outcomes between the two intervention groups will be compared using the Chi-square test. The relative risk reduction, absolute risk reduction and the number needed to treat with the permissive underfeeding diet to prevent one death will be calculated. Moreover, the mechanical ventilation duration and ICU and hospital LOS in the two arms will be compared using the independent Student’s t-test. For binary outcomes, logistic multivariate regression will be used to test the intervention effect, controlling for covariates when appropriate, such as when analyzing subgroups. Adjusted relative risks and associated 95% CIs will be estimated using Cox proportional hazards regression modeling. Primary outcome will also be analyzed using survival analysis. All tests of significance will be at the 5% significance level, and two-sided. Analyses will be conducted using SAS version 9.2 and/or SPSS.

#### Subgroup analyses

The following are *a priori* subgroups that will be analyzed: non-operative and operative patients, diabetic and non-diabetic patients, APACHE II ≤18 and >18, specific admission diagnoses (sepsis and traumatic brain injury), patients on vasopressors at baseline and those who are not and inclusion blood glucose (categorized according to the median value).

### Monitoring for safety

Several measures are taken to minimize, observe and document any potential safety concerns. First, serious adverse events (SAEs) will be reported immediately to the Steering Committee, IRB and DMC. Second, an independent DMC will be monitoring the safety of the trial. Third, one interim analysis has been conducted, and the next one will be done once 2/3 of the total sample size is recruited. Finally, site visits to the participating centers by the principal investigator or designee are planned for data monitoring including data verification and review of the consent process.

## Discussion and Trial status

This trial was designed in 2008. The protocol passed through multiple amendments. Final approval from IRB was obtained on 8 April 2009. The study began enrolling at the principal site in November 2009. It was subsequently joined by one national (King Faisal Specialist Hospital and Research Center) and three international centers (Ottawa Hospital, General Campus, the Ottawa Hospital, Civic Campus and Mount Sinai Hospital in Toronto). The expected duration of the study is four years. The first DMC meeting was held on 7 October 2009 and a follow-up meeting on 9 February 2011. The study monitor visited the coordinating site on 7 January 2012 and submitted a detailed report to the sponsor. The first interim analysis was carried out in March 2012 as soon as 287 patients (one-third of the sample size) were completed with 90-day outcome. Unblinded results were submitted to the DMC, who indicated that trial recruitment should proceed. The results were maintained blinded to the investigators/research team with the exception of the biostatistician.

## Abbreviations

ACCEPT: Algorithms for Critical-Care Enteral and Parenteral Therapy; APACHE: Acute Physiology and Chronic Health Evaluation; ASPEN: American Society for Parenteral and Enteral Nutrition; BMI: body mass index; DMC: Data Monitoring Committee; ICH-GCP: International Conference on Harmonization-Good Clinical Practice guidelines; IRB: Institutional Review Board; KAIMRC: King Abdullah International Medical Research Center; KSAUHS: King Saud bin Abdulaziz University for Health Sciences; LOS: Length of Stay; PN: Parental Nutrition; RCT: Randomized Control Trial; SCCM: Society of Critical Care Medicine.

## Competing interests

This trial is funded by KAIMRC/KSAUHS, Riyadh, Saudi Arabia. The sponsor had no influence on the design of the protocol, patient recruitment or data generation and will not have any impact on the analysis of the results or writing of the manuscript. The authors have no financial or non-financial competing interests to declare.

## Authors’ contributions

YMA participated in the design of the trial, statistical analytical plan, database development and in writing the manuscript; SHH contributed to the design of the trial, randomization of the patients and editing the manuscript; ASA contributed to the design of the trial, randomization of the patients and drafting the manuscript; HAD contributed to the design of the trial, discussing the consent with patients’ surrogates, and editing the manuscript; HMT helped in the design, statistical analysis plan and critical revision of the manuscript; MS participated in the data collection, protocol implementation and critical revision of the manuscript; GJ contributed to the drafting and critical revision of the manuscript, LM contributed to the drafting and critical revision of the manuscript; SM contributed to the drafting and critical revision of the manuscript; OS contributed to the drafting and critical revision of the; MS contributed to randomization, data acquisition, data entry and quality check, and critical revision of the manuscript; LA contributed to randomization, data acquisition, data entry and critical revision of the manuscript; BS contributed to randomization, data acquisition, data entry and critical revision of the manuscript. All the authors read and approved the final manuscript.

## Sponsor

King Abdullah International Medical Research Center (KAIMRC), Riyadh, Saudi Arabia.

## Supplementary Material

Additional file 1 Appendix A, Appendix B. Click here for file
