# Supplementary material for: Permissive underfeeding versus target enteral feeding in adult critically ill patients (PermiT Trial): a study protocol of a multicenter randomized controlled trial
Source: Trials. 2012 Oct 12;13:191. doi: 10.1186/1745-6215-13-191 (PMC3517534; doi:10.1186/1745-6215-13-191)
Supplement: Additional file 1 — Appendix A, Appendix B. [file 1745-6215-13-191-S1.pdf]

APPENDIX A

Steps of calculation of caloric requirement

Mechanically ventilated patients with BMI < 30: Penn State Equation

Step 1) The Harris-Benedict Equation for Basal Energy Expenditure (BEE)(1)

For men: BEE = 66.5 + 13.8(W) +5.0(H) – 6.8(A)

For women: BEE = 655.1 +9.6(W) +1.9(H) – 4.7(A)

(W =weight in kilograms; H = height in cm; A = age in years)

Step 2) Penn State Equation (2003a version)(2)

Resting Metabolic Rate = Basal Metabolic Rate (0.85) + (VE) (33) + Tmax (175) – 6433.

(Basal Metabolic Rate calculated using the Harris-Benedict equation, minute ventilation (VE) in liters per min (L/min), and maximum temperature (Tm ax) in degrees Celsius).

Mechanically ventilated patients with BMI ≥ 30: Ireton-Jones, 1992 Equation (3)

Ventilator dependent IJEE (v) = 1925 - 10 (A) + 5 (W) + 281 (S) + 292 (T) + 851(B)

Spontaneously breathing patients: Ireton-Jones, 1992 Equation(3)

Spontaneously breathing IJEE (s) = 629 - 11 (A) + 25 (W) - 609 (O)

{The Ireton-Jones Equations use age (A) in years, body weight (W) in kilograms (kg), sex (S, male = 1, female = 0), diagnosis of trauma (T, present = 1, absent = 0), diagnosis of burn (B, present = 1, absent = 0), obesity: BMI ≥ 30}.

Appendix B

Primary Outcome: 90 day-all cause mortality.

Secondary Outcomes:

- ICU mortality: death in the ICU during the same ICU admission.
- Hospital mortality: death in the hospital (in ICU or on ward) during the same hospital admission
- 180 day mortality: death before or at day 180 of enrollment.
- Sequential Organ Failure Assessment (SOFA) scores recorded on days 1,3,7, 14, 21 and 28

Tertiary Outcomes:

- ICU LOS: Number of calendar days between ICU admission and ICU discharge.
- Hospital LOS: Number of calendar days between hospital admission and hospital discharge.
- Duration of mechanical ventilation: Number of calendar days between start and end of mechanical ventilation.
- Hypoglycemia: The occurrence of low blood glucose of $\leq$2.2 mmol/L.
- Incidence of health care-associated infections including ventilator-associated pneumonia, blood stream infections, urinary tract infection.
- Health care-associated sepsis: The presence of systemic inflammatory response syndrome and either positive microbiology cultures and/or clinical signs of infection 48 h after ICU admission.
- Refeeding Syndrome is defined as the biochemical derangements occurring after initiating nutritional support with fall in potassium < 2.8 mmol/l, magnesium <0.66 mmol/l or phosphate <0.70 mmol/l.
- Diarrhea is defined as per the World Health Organization, as having 3 or more loose or liquid stools per day.

References

1. Harris JA, Benedict JA. Biometric studies of basal metabolism in man. Carnegie Institute of Washington, DC; 1919: Publication no.270. 1919.
2. Frankenfield D, Smith JS, Cooney RN. Validation of 2 approaches to predicting resting metabolic rate in critically ill patients. JPEN J Parenter Enteral Nutr. 2004;28(4):259-64.
3. Ireton-Jones CS, Turner WW, Jr., Liepa GU, Baxter CR. Equations for the estimation of energy expenditures in patients with burns with special reference to ventilatory status. J Burn Care Rehabil. 1992;13(3):330-3.
